# A Brief Educational Intervention Improves Medication Safety Knowledge in Grandparents of Young Children

**DOI:** 10.3934/publichealth.2015.1.44

**Published:** 2015-03-11

**Authors:** Maneesha Agarwal, Janice Williams, Demetrios Tavoulareas, Jonathan R. Studnek

**Affiliations:** 1 Pediatric Emergency Medicine, Emory University & Children's Healthcare of Atlanta, Atlanta, GA 30322, USA; 2 Department of Emergency Medicine, Carolinas Medical Center, Charlotte, NC 28203, USA; 3 Emergency Medicine Department, ApolloMD, Miami, FL, USA; 4 Mecklenburg EMS Agency, Charlotte, NC, USA

**Keywords:** poisoning, prevention and control, overdose, child, safety

## Abstract

**Background and Objectives:**

Increasing grandparent-grandchild interactions have not been targeted as a potential contributing factor to the recent surge in pediatric poisonings. We hypothesized that in grandparents with a young grandchild, a single educational intervention based on the PROTECT “Up & Away” campaign will improve safe medication knowledge and storage at follow-up from baseline.

**Methods:**

This prospective cohort study validated the educational intervention and survey via cognitive debriefing followed by evaluation of the educational intervention in increasing safe medication storage. Participants had to read and speak English and have annual contact with one grandchild ≤ 5-years-old. Participants were recruited from a convenience sample of employees in a regional healthcare system. They completed a pre-intervention survey querying baseline demographics, poisoning prevention knowledge, and medication storage, followed by the educational intervention and post-intervention survey. Participants completed a delayed post-intervention survey 50–90 days later assessing medication storage and poisoning prevention knowledge. Storage sites were classified as safe or unsafe *a priori*; a panel classified handwritten responses.

**Results:**

120 participants were enrolled; 95 (79%) completed the delayed post-intervention survey. Participants were predominantly female (93%) and white (76%); 50% had a clinical degree. Participants averaged 1.9 grandchildren. Initially, 23% of participants reported safe medication storage; this improved to 48% after the intervention (OR 6.4; 95% CI = 2.5–21.0). 78% of participants made at least one improvement in their medication storage after the intervention even if they did not meet all criteria for safe storage. Participants also demonstrated retention of poisoning prevention knowledge.

**Conclusions:**

This brief educational intervention improved safe medication storage and poisoning prevention knowledge in grandparents of young children; further evaluation of this intervention is warranted.

## 1. Introduction

Every year, more than 70,000 children are evaluated in emergency departments for unintentional exposures related to medicines [1]. Despite decades of public health efforts related to poisoning prevention, this number is increasing. A recent study documented a 43% increase in pediatric poisoning injuries related to pharmaceutical agents reported to the National Poison Data System (NPDS) from 2001 to 2008 with similar increases in admission, emergency department use, and exposures [2].

The growing numbers of Americans on prescription medications may be a contributing factor to the pediatric poisoning epidemic [3],[4]. This may include grandparents, who are having more contact with their grandchildren than ever before [5],[6]. These older generations are more likely to use prescription medicines that may be fatal, even in small doses, such as beta-blockers and calcium channel blockers [7],[8]. Additionally, grandparents may have knowledge gaps with regards to child safety [9] and store medicines within easy reach of children [10].

Given the increasing interactions between grandparents and grandchildren in the context of the growing number of pediatric poisonings with pharmaceuticals, easily replicable interventions must be implemented to impact this public health concern. Prior interventions focused on child resistant containers were somewhat effective in decreasing pediatric pharmaceutical poisonings [11],[12]; however, more recent literature exposes their imperfections and fallibility [13]. Complementary education initiatives may be helpful. Basic safety education as brief as 5-minutes duration may improve poisoning-prevention practices [14],[15]. Unfortunately, older educational programs are compromised by outdated recommendations, such as use of syrup of ipecac [16] and have targeted delivery in home visitation programs[17],[20],[26], primary care sites [21]–[24], and emergency departments [25]. The messages contained in the Preventing Overdoses and Treatment Exposures Task Force's (PROTECT) “Up & Away” campaign––a multi-media approach that advocates storing medicines out of reach and out of sight of young children1––offer a potential basis for an effective safety education program. The messages are current, amenable to use in multiple settings, and make use of current technologies and media. Many pediatric poisoning pharmaceutical exposures could be avoided by simple adoption of the “Up & Away” message [27].

Therefore, we hypothesize that an educational intervention based on the “Up & Away” campaign will promote positive change in grandparents' knowledge of pediatric poisoning prevention and storage of their medication. The purpose of this study was to assess whether a single educational session could improve safe medication storage based on self-report 50–90 days after the educational session in a cohort of grandparents with at least annual contact with a grandchild ages 5-years-old or younger.

## 2. Methods

For this prospective cohort study, researchers recruited a convenience sample from employees within a regional hospital system. Researchers used flyers, advertisements on the employee intranet, e-mail messages, and word of mouth for recruitment. Prior to the study, participants only knew that the study involved their role as grandparents with their grandchildren. Participants had to possess fluency and literacy in conversational English and have at least one grandchild 5-years-old or younger that they interacted with at least annually. This study was approved by the Carolinas HealthCare System Institutional Review Board.

Because there were no known validated tools for assessing grandparent-grandchild interactions, basic poisoning prevention knowledge, or medication storage, surveys were developed and validated using consensus opinion of the study authors followed by cognitive debriefing on 12 participants [28]. Based on cognitive psychology, this methodology involves structured interviews and questions to minimize misinterpretation of survey questions while enhancing memory recall of participants. Cognitive debriefing was also used to validate our educational intervention, a 15-minute, scripted PowerPoint (Microsoft Corp., Redmond, WA) based on the “Up & Away” campaign. Key points of this educational intervention included the worsening epidemiology of pediatric poisonings, developmentally appropriate child behavior that results in poisonings, grandparents' role in prevention, appropriate actions in case of a poisoning, and safe storage of medications. Individuals who participated in our cognitive debriefing were not eligible for inclusion in the larger study or data analysis. There were no significant changes to the survey or educational intervention after cognitive debriefing.

After validation of the study materials, additional participants were recruited and convened in small groups of 2–12 participants. During the one hour session, they completed the pre-intervention survey, participated in the educational intervention led by one of the study authors (JW or MA), and completed the immediate post-intervention survey. Incentives included a free meal during the session, a child-based poisoning prevention activity book, and a magnet with the phone number for the local poison control center.

Participants completed a delayed post-intervention survey via phone 50–90 days after the educational intervention. Follow-up was conducted by a research assistant as a blinded surveyor to add to the study design. Three attempts were made to contact Participants via telephone followed by two e-mails, each separated by 48–72 hours. Participants who did not respond within 90 days of enrollment were considered lost to follow up.

Unsafe storage of medication at either the grandparents' home or the grandchild's home was the primary outcome variable of interest. Storage sites were classified as unsafe if they met any of the criteria found in Table 1. The survey specifically queried medication storage in the kitchen, bathroom, and bedroom in the grandparents' and grandchildren's homes; there was also a free response section for other potential storage sites in each room and to account for other potential locations within the homes (i.e., linen closet). Handwritten responses were analyzed independently by three authors (MA, DT, JW) and dichotomized as either safe or unsafe with simple majority determining categorization. If insufficient detail was provided, responses were categorized as unsafe. A group of five questions related to basic poisoning prevention knowledge was a secondary variable of interest, described in Table 2. All responses were analyzed in a dichotomous fashion.

**Table 1.** Criteria for safe versus unsafe storage.

Unsafe storage defined as:						
1.	Medicines in the kitchen NOT stored in cabinet above countertop OR NOT in drawer/cabinet secured with child safety lock.					
2.	Medicines in the bathroom NOT stored in cabinet above countertop OR NOT in drawer/cabinet secured with child safety lock.					
3.	Medicines in the bedroom NOT stored on shelving or surface above shoulder level OR NOT in drawer/cabinet secured with child safety lock.					
4.	Handwritten responses indicating storage is on shelving located at surface or below shoulder level OR NOT in drawer/cabinet secured with child safety lock. OR responses without adequate detail to determine safe storage					

**Table 2.** Basic Poisoning Prevention Knowledge.

	Before Intervention	Immediately After Intervention	Delayed 50-90 Days After Intervention	OR for Delayed (95% CI)
“Poisoning in young children is becoming a bigger problem.”	76%	98%	95%	7.3 (2.2–38.3)
“A child-resistant container means it is completely child-proof.”	77%	93%	94%	16.0 (2.5–671.0)
“One pill of a medicine can kill a child.”	82%	92%	99%	15.0 (2.3–631.5)
“If you think a child may have gotten into a medicine AND they do not have any symptoms, the best thing to do is”				
Call RN/MD	7%	0%	0%	
Call poison control	76%	96%	91%	
Call 9-1-1	8%	3%	5%	
Go to ER/Urgent Care	8%	2%	4%	
“Do you have the Poison Center number readily available?”	37%		95%	57.0 (9.8–2290.4)

All tested questions were statistically significant with *p* < 0.0125 corrected for multiple testing.

Other independent variables collected related to baseline demographics, interactions with grandchildren, and medication usage. Collected demographics included age, gender, race, and level of education. Given participant characteristics, race was analyzed as a dichotomous value between white and non-white. Education was assessed primarily related to the possession of a clinical degree for potential bias indication. Number of grandchildren 5-years-old or younger per participant was analyzed to determine a mean number and range of children per participant. Co-residence with a grandchild was analyzed as a dichotomous value. Frequency of visitation with grandchildren was dichotomized to at least weekly versus less frequent interaction. The number of medications taken daily, medicines in the participant's residence, and use of specific storage devices (pill organizers, sandwich baggies, exclusive storage in pill bottles) were analyzed as multiple, mutually exclusive categories. Traveling with medicine, prior experiences with a poisoning, and intent to change medication storage habits were evaluated as dichotomous variables.

Prior literature suggested that 20% of our cohort would store medication safely before the educational intervention [10]. A sample size of 400 participants would allow for a 25% loss to follow up while allowing this study to demonstrate a 50% improvement in medication storage with 80% power and a set alpha of 0.05. Calculated a priori, even with a sample size of 100, this study could detect at least a 90% improvement in medication storage.

Data collected from the pre-intervention survey, immediate post-intervention survey, and delayed post-intervention survey were entered into an Excel (Microsoft Corp. Redmond, WA) spreadsheet, then transferred to a STATA (StataCorp, College Station, TX) database for analysis. We conducted descriptive statistical analysis on baseline demographics and used the Chi-Squared McNemar's Test to assess for differences in safe storage before and after the intervention. Results are reported as frequencies and odds ratios with associated 95% confidence intervals. This methodology was also used to analyze secondary outcomes including changes in poisoning prevention knowledge across all 3 survey time-points and changes in safe storage in individual locations (ex: participants' homes, grandchildren's homes, participants' kitchen, etc). Bonferroni correction was applied whenever there was multiple testing.

## 3. Results

Over 6 months, 120 participants were enrolled; and 95 (79%) of these participants responded to the delayed post-intervention survey 50?90 days later. There were no significant differences between those who enrolled in the study and those who completed the protocol. The participants were predominantly Caucasian (76%) and female (93%) with most having education beyond high school (89%). Fifty percent had clinical degrees, and there were no physicians. Sixty-two percent of participants had weekly or more frequent interactions with their grandchildren in their own home versus 44% in the grandchild's home; 18% resided with a grandchild. Twenty-eight percent of participants took more than 5 different medications daily, and 57% of participants had more than 10 medicine bottles in their home. Fifty-one percent of participants used pill organizers and 71% regularly traveled with medicine on their person (i.e., in a pocketbook). Additionally, 71 participants endorsed taking medicines with them to a grandchild's home. (Table 3)

**Table 3.** Demographics/Grandchild Interactions/ Medication Use and Storage of Recruited Participants.

All participants, *N* = 120		
Age (years), mean, standard deviation	55.7, 6.4	
Female	111/120	93%
White race	91/120	76%
Education * multiple degrees were indicated by some clinical added to college attainment.		
General Educational Development/high school diploma	13/120	11%
Some college, no degree	35/120	29%
Associate degree	17/120	14%
College degree	33/120	28%
Post-graduate degree	22/120	18%
Clinical degree (Registered Nurse, Pharmacy Tech, etc.)	60/120	50%
Grandchild interactions		
Grandchildren/participant, mean, range	1.9, 1–7	
Resides with grandchild	21/119	18%
At least weekly grandchild visitation in own home	73/118	62%
At least weekly grandchild visitation in grandchild's home	50/114	44%
Medication availability		
Medicines taken daily		
1–2 different medicines	38/109	35%
3–5 different medicines	41/109	38%
6–10 different medicines	26/109	24%
11+ different medicines	4/109	4%
Medicine bottles in home		
1–2 bottles	5/118	4%
3–5 bottles	19/118	16%
6–10 bottles	27/118	23%
11–15 bottles	31/118	26%
16+ bottles	36/118	31%
Storage devices		
Uses pill organizers for medicines	61/119	51%
Uses baggies for medicines	7/119	6%
Exclusive storage of medicines in pill bottles	51/119	43%
Travels with medicine	80/112	71%
Medication Storage		
In own home		
Kitchen	95/120	79%
Bathroom	73/120	61%
Bedroom	39/117	33%
In grandchild's home		
Kitchen	71/108	66%
Bathroom	90/108	83%
Bedroom	68/107	64%
Prior experience with a poisoning	21/119	18%
Expressed intent to change storage on immediate post-intervention survey	107/120	89%

At the time of enrollment, 23% of participants reported criteria for safe storage in both locations of their own homes and in their grandchildren's homes (Table 4 and 5). When assessed 50–90 days after the intervention, safe storage was improved to 48% (*p* < 0.0001). Post-intervention, grandparents were more likely to report safe medication storage at both their home and their grandchild's home as compared to pre-intervention. There was statistically significant improvement in reported medication storage overall in the participants' homes (*p* < 0.0001) and grandchildren's homes (*p* = 0.0003). Participants reported statistically significant improvements in safe storage in their own homes in the kitchen (*p* < 0.0001), bathroom (*p* < 0.0003), and bedroom (*p* = 0.0006). Notably, the only room in grandchildren's homes with statistically significant improvement in reported safe storage was the bedroom (*p* = 0.0093) compared to the kitchen (*p* = 0.2752) and bathroom (*p* = 0.0956). Seventy-four of 95 (78%) participants who completed the post-intervention survey reported at least one positive change in medication storage, defined as reporting the movement from unsafe categorization to safe categorization in at least one room (i.e., the grandchild's kitchen). Moving medicines from a countertop to shelving above shoulder level was a commonly noted improvement in over 30 participants; 21 participants also noted storage of medicines in locked vehicles when visiting grandchildren. Interestingly, 40 participants reported medication storage in a purse; this does not include related mentions of storage in luggage, gym bags, golf bags, suitcases, and carry-ons. Participants also demonstrated significant acquisition of basic poisoning prevention knowledge (Table 2) in all queried domains in both the immediate and delayed post-intervention surveys.

**Table 4.** Safety of Medication Storage in Participants' Homes.

	Pre-Intervention Safe Storage	DelayedPost-Intervention Safe Storage	*P*-value
ALL Locations*	23%	48%	< 0.0001
Participants'	38%	67%	
Home*			< 0.0001
Kitchen*	69%	91%	< 0.0001
Bathroom*	80%	94%	= 0.0003
Bedroom*	78%	91%	= 0.0006

*Indicates statistically significant *p* < 0.0055, corrected for multiple testing

**Table 5.** Safety of Medication Storage in Grandchildren's Homes.

	Pre-Intervention Safe Storage	DelayedPost-Intervention Safe Storage	*P*-value
Grandchild's Home*	43%	58%	= 0.0003
Kitchen	80%	80%	= 0.2752
Bathroom	92%	97%	= 0.0956
Bedroom**	78%	89%	= 0.0093

*Indicates statistically significant *p* < 0.0055, corrected for multiple testing **Delayed post-intervention data on bedroom storage was missing from one participant

## 4. Discussion

In this study, an educational intervention resulted in a reported doubling of safe medication storage among grandparents with active visitation with grandchildren ages 5-years-old or younger 50?90 days after the intervention. Statistically significant improvement in medication storage was reported overall as well as in the grandparents' and the grandchildren's homes; however, changes were more robust in the grandparents' home. This may reflect a greater ability to control one's own environment or a greater investment in one's own home. While 48% of participants ultimately met criteria for safe storage of medication in all locations, nearly 80% of participants reported some level of positive change with regards to medication storage. While poisoning prevention knowledge was already robust in our participants, perhaps secondary to recruitment from employees in a healthcare setting, this was further enhanced and retained 1–3 months after the intervention.

Pediatric exposures are increasing [1],[2],[28]; based on the aging population, rising use of prescription medications [3],[29], and increasing interactions between grandparents and grandchildren, grandparents may be a contributing factor [30],[31]. Data analysis from the United States Consumer Product Safety Commission's National Electronic Injury Surveillance System implicated grandparents as the source of a medicine related poisoning resulting in an emergency evaluation in 38% of cases [32]; this is further supported by the strong correlation between pediatric poisonings with the increasing use of prescription medicines in adults [4]. Additionally, grandparents are likely missing injury prevention messages delivered during routine health care exchanges or relying on older injury prevention messages and parenting memories rather than current recommendations [9],[31],[33]. Thus, effective, grandparent-targeted interventions, such as the educational intervention in this study, may contribute to the reduction of harmful pediatric poisonings. Besides community based programs for education, which this study shows may receive less than optimal attendance, healthcare providers may look to this educational material for their patients to provide some education, since patients presenting with a possible pediatric poisoning, have not in the past received poison prevention materials [33].

Prior studies suggest that education can improve knowledge and potential behavior change regarding safe medication storage and poisoning prevention knowledge among families and even children; yet many of these older studies are compromised by outdated recommendations and in specific environments requiring extensive resources. Additionally, no studies to date have evaluated the impact of an educational intervention targeted at grandparents. The strength of this study lies in the use of an educational intervention that can be delivered in multiple settings using current technology and recommendations while impacting a specific target population, grandparents. For example, participants were encouraged to make use of their current technology by programming the poison control center number into their cell phones. This study also provides further knowledge regarding grandparent-grandchild interaction, such as frequency of visits, as well as medication storage habits. In fact, nearly 20% of our participants reported co-residence with a grandchild 5-years-old or younger, representing constant exposure to a poisoning source.

A fifteen minute educational intervention is a bit long considering possible embedment of messages during clinical encounters, grandparent classes, and other venues; however, even 5-minutes of education regarding poisoning prevention may be effective, and further study of a potentially further shortened intervention is warranted [14]. Studies have demonstrated that brief poisoning prevention videos and use of a computer based kiosk on injury prevention while families wait for medical care effectively improve knowledge and could serve as a model for future study of this “Up & Away” educational intervention [22],[34],[35]. Targeting adults who are filling prescriptions for medicines that may be lethal in small doses may also be efficacious, as pharmacists infrequently advise older adults about poisoning prevention [36]. While costly, a public relations outreach may also be efficacious as demonstrated in a regional poison control center study [37]. Community volunteers may even be useful in further up scaling of this intervention with minimal training as documented in other injury prevention initiatives [15],[25].

Thus, further study of the “Up & Away” educational intervention is merited – in various settings, in abbreviated format, and with varying target populations. Qualitative data and comments from the cognitive debriefing and formal study provide insight in how best to simplify this educational intervention. For example, at least 40 unique participants identified storing medicines in a pocketbook equivalent, not inclusive of related traveling bags; discussion about purses as a potential source of poisoning seemed to resonate with many participants who had not previously considered this. Additionally, while participants were aware of poison control centers, many did not have the number readily available; yet many were noted to promptly program the number into their cell phones when suggested by the educational intervention. Other particularly impactful motivators of change included discussion of child development, inability to distinguish between candy and medicine, lethality of some pills in single doses, and discussion of how child resistant containers are not child proof.

A major limitation of this study is that participants came from a sample unrepresentative of the general US population of grandparents given their ongoing employment in a health care setting; further study among varied populations is certainly warranted. Additionally, a larger sample size would allow for further sub-group analysis. However, this highly motivated sample that may arguably possess better than average health literacy still demonstrated a statistically significant improvement in their medication storage and poisoning prevention knowledge. Reliance on volunteers may have resulted in recruitment of participants that were already highly motivated to make changes to positively impact their grandchildren. Also, while positive changes in medication storage were demonstrated 50–90 days after the intervention, these health behavior changes and improvements in basic poisoning prevention knowledge may decline over time. Finally, it is unclear whether behavior change in safe medication storage translates into reduced pediatric poisonings from medicines. However research suggests proving an intervention makes a statistically significant impact on pediatric poisoning exposures would require over 21,500 person-years per arm [16].

Data analysis relied upon self-reported survey responses, making this study prone to recall bias and social desirability bias. However, verifying storage of medicines with home visits would have been cost- and time-prohibitive, yet still fraught with the potential for participants to alter medication storage in anticipation of a study coordinator's visit. Additionally, studies have found high positive predictive values for the safe storage of medication as reported on a survey and subsequently validated on a home visit, suggesting that home visits may add less value than assumed [38],[39]. There may have been a potential bias as well if the 79% of participants who completed the delayed post-intervention survey were more proactive than those who did not follow up. However, basic demographics did not demonstrate any statistically significant difference between the two groups and we used a robust methodology to retain and contact participants as described in our protocol.

## 5. Conclusion

The growing burden of pediatric poisonings from medicines requires further efforts from the public health community to address this worsening problem. This study suggests that a simple, brief, easily reproducible educational intervention based on current recommendations and technology can significantly improve medication storage and basic knowledge regarding poisonings in grandparents of young children and serve as a valuable piece of poisoning prevention along with other measures, such as child-resistant packaging. There are many possibilities for further study and deployment of this educational intervention based on the PROTECT initiative's “Up & Away” campaign.
